# A Radio-genomics Approach for Identifying High Risk Estrogen Receptor-positive Breast Cancers on DCE-MRI: Preliminary Results in Predicting Onco*type*DX Risk Scores

**DOI:** 10.1038/srep21394

**Published:** 2016-02-18

**Authors:** Tao Wan, B. Nicolas Bloch, Donna Plecha, CheryI L. Thompson, Hannah Gilmore, Carl Jaffe, Lyndsay Harris, Anant Madabhushi

**Affiliations:** 1 School of Biological Science and Medical Engineering, Beihang University, Beijing, China; 2 Dept. of Biomedical Engineering, Case Western Reserve University, OH, USA; 3 Dept. of Radiology, Boston University School of Medicine, MA, USA; 4 Dept. of Radiology, University Hospitals Case Medical Center, OH, USA; 5 Dept. of Nutrition, Case Western Reserve University, OH, USA; 6 Division of Anatomic Pathology, Case Western Reserve University, OH, USA; 7 Division of Hematology and Oncology, Seidman Cancer Center, OH, USA

## Abstract

To identify computer extracted imaging features for estrogen receptor (ER)-positive breast cancers on dynamic contrast en-hanced (DCE)-MRI that are correlated with the low and high Onco*type*DX risk categories. We collected 96 ER-positivebreast lesions with low (<18, *N* = 55) and high (>30, *N* = 41) Onco*type*DX recurrence scores. Each lesion was quantitatively charac-terize via 6 shape features, 3 pharmacokinetics, 4 enhancement kinetics, 4 intensity kinetics, 148 textural kinetics, 5 dynamic histogram of oriented gradient (DHoG), and 6 dynamic local binary pattern (DLBP) features. The extracted features were evaluated by a linear discriminant analysis (LDA) classifier in terms of their ability to distinguish low and high Onco*type*DX risk categories. Classification performance was evaluated by area under the receiver operator characteristic curve (Az). The DHoG and DLBP achieved Az values of 0.84 and 0.80, respectively. The 6 top features identified via feature selection were subsequently combined with the LDA classifier to yield an Az of 0.87. The correlation analysis showed that DHoG (*ρ* = 0.85, *P* < 0.001) and DLBP (*ρ* = 0.83, *P* < 0.01) were significantly associated with the low and high risk classifications from the Onco*type*DX assay. Our results indicated that computer extracted texture features of DCE-MRI were highly correlated with the high and low Onco*type*DX risk categories for ER-positive cancers.

In the United States, approximately 70% of all breast cancer patients are diagnosed with estrogen receptor (ER)-positive primary tumors, and many of these women will receive hormonal therapy and adjuvant chemotherapy^1^,^2^. Identifying patients who can be treated with hormonal therapy alone would limit toxicity to those most likely to benefit^3^. Currently, the selection of individualized therapy for patients with ER-positive, human epidermal growth factor receptor type 2 (HER2)-negative early stage breast cancers is guided by the Onco*type*DX gene expression assay (Genomic Health Inc., Redwood City, CA)^4^, which produces a recurrence score between 0–100 to predict the likelihood of disease recurrence with tamoxifen and improves the oncologist’s ability to estimate benefit from adjuvant hormonal and chemotherapy^5^,^6^. However, the Onco*type*DX assay is performed on tissue biopsy specimens and involves additional tumor handling and shipping of tissue, and delays in time to treatment, all of which add costs and anxiety for patients^3^,^7^.

Computer-aided diagnosis (CAD) systems have been previously used with dynamic contrast enhanced magnetic resonance imaging (DCE-MRI) of the breast to predict the presence or absence of cancer^8^. These CAD tools aim to increase the accuracy of diagnosis of breast cancer on MRI while also aiming to reduce inter-observer variability. However, more recently a number of groups have begun to focus on identifying computer extracted features on MRI that might be associated with underlying biology molecular subtype and risk of recurrence of the tumor^9^,^10^,^11^. A radiogenomic approach presented by Yamamoto *et al.*^12^ found a significant correlation between breast MRI (1.5 Tesla) features and a number of important breast cancer related gene sets. Correlation studies by Vassiou *et al.*^13^ and Chang *et al.*^14^ showed that DCE-MRI (1.5 Tesla) based imaging features, such as tumor margin, enhancement pattern, and kinetic characteristics, were associated with pathological prognostic factors for the prediction of clinical outcome during treatment of breast cancer. A recent study conducted by Sutton *et al.*^15^ showed that two MRI (1.5 or 3.0 Tesla) derived statistical image features were significantly correlated with the median Onco*type*DX recurrence scores with a range of 0–45. Ashraf *et al.*^9^ presented a method for identifying correlation between computer-extracted morphologic and kinetic features from DCE-MRI sequences obtained at a 1.5T magnet with validated prognostic gene expression profiles of breast cancers. In addition, Agner *et al.*^8^ presented an approach called textural kinetics (TK), which involved measuring dynamic changes in breast lesion texture during contrast uptake. These TK features were able to separate different molecular subtypes of breast cancers (triple negative, ER-positive, HER2-positive, fibroadenoma) on DCE-MRI obtained at either 1.5T or 3T^16^.

In this work, we investigate the ability of TK features on DCE-MRI to distinguish ER-positive breast cancers between low and high Onco*type*DX risk categories (i.e., Onco*type*DX recurrence score <18 and Onco*type*DX recurrence score >30). Our approach is different from the work presented by Ashraf *et al.*^9^, in which dynamic features were computed based on the estimation of parameters on time-intensity curve, e.g., peak enhancement, wash-in and wash-out slop. Our approach is also different from the work published by Sutton *et al.*^15^, in which the image features (i.e., morphological, static first-order, and Haralick texture features) were extracted from breast lesions on pre- and three post-contrast MR images. In this study, we focus on TK features that allow for characterization of dynamic texture changes, specifically texture involving dynamic histograms in tumors on 1.5 Tesla DCE-MRI. Also our TK features are different from the approach by Agner *et al.*^8^,^16^, in that it involves quantification of kinetic texture in a new way - dynamic histogram of oriented gradients (DHoG) and dynamic local binary patterns (DLBP).

The histogram of oriented gradients (HoG)^17^, local binary patterns (LBP)^18^, and their spatio-temporal representations^19^,^20^ are image texture features that have been previously employed for detecting breast masses on mammographic images^21^,^22^. Unlike the approach in Agner *et al.*^8^,^16^ which attempted to fit a single parametric curve to explain the changes in lesion texture during the contrast wash-in and wash-out, the DHoG and DLBP approaches allow for construction of a unique lesion signature that captures the frequency of occurrence of different spatio-temporal textural patterns.

In this study, we evaluate the ability of the DHoG and DLBP features extracted from DCE-MRI at 1.5 Tesla, via a linear discriminant analysis (LDA) classifier to distinguish low and high risk ER-positive breast cancers, risk having been established via the Onco*type*DX assay.

## Results

**Figure 1:**
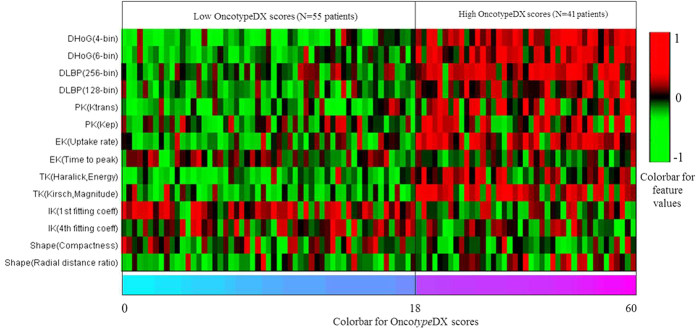
Heat map showing the values of best two identified features in each feature class (DHoG, DLBP, PK, EK, TK, IK, shape). The columns represent breast tumors and rows represent features. DHoG = dynamic histogram of oriented gradient; DLBP = dynamic local binary pattern; PK = pharmacokinetics; EK = enhancement kinetics; TK = textural kinetics; IK = intensity kinetics.

Table 1 shows the best two identified features in each feature class (shape, PK, EK, IK, TK, DHoG, DLBP) associated with Az, PPV, NPV, *ρ* (correlation coefficient), RSD, and *err* (error rate of classification). The numbers of bins used in the DHoG and DLBP features were {2, 4, 6, 8, 10} and {8, 16, 33, 64, 128, 256}, respectively. Higher *ρ* value indicates a stronger relationship between risk stratification via the features and Onco*type*DX. The post-hoc power analysis revealed no significant difference between features identified from imaging data acquired at Sites I and II which were found to discriminate high versus low Onco*type*DX risk categories. The heat map (Fig. 1) shows the values of all the features listed in Table 1 for all the patient studies. The values of best two identified features in each feature class and the six top performing features obtained from the LDA based feature selection method are listed in Supplementary Table S1 online.

### Shape Features for Discriminating ER-positive Breast Cancers

The feature compactness used to measure the speculation of tumor margin yielded the best discriminability among the computerized shape features. The lower values of compactness for the high Onco*type*DX breast cancers (−20.76 ± 7.32) compared with the low Onco*type*DX cancers (−19.05 ± 9.14) appears to suggest that higher Onco*type*DX score cancers tend to be associated with more speculation compared to cancers with low Onco*type*DX risk scores. Table 1 appears to suggest that shape features are less useful for differentiating between low and high Onco*type*DX risk score ER-positive lesions compared to pharmacokinetic and textural kinetic features (i.e., PK, EK, IK, TK, DHoG, and DLBP).

### PK Features for Discriminating ER-positive Breast Cancers

**Figure 2:**
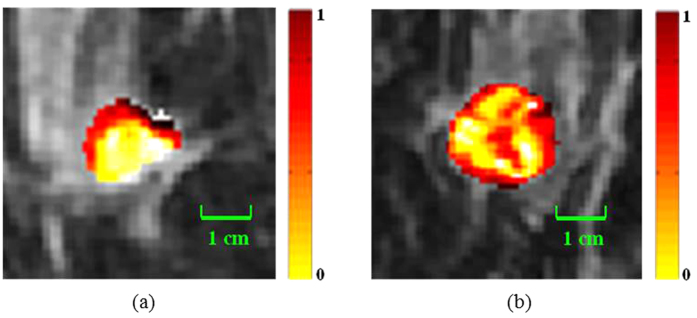
Comparison of pharmacokinetic feature (K^*trans*^) of estrogen receptor (ER)-positive breast lesions with low and high Onco*type*DX recurrence scores. (**a**) K^*trans*^ in 47-year-old women with low Onco*type*DX (=8), low grade ER-positive breast lesion, and (**b**) K^*trans*^ in 54-year-old women with high Onco*type*DX (=58), high grade ER-positive breast lesion. The K^*trans*^ values are encoded in a color scale, where large values are represented in dark red and small values are represented in yellow. Note a greater heterogeneity within the high risk ER-positive breast cancers compared to low risk breast cancers.

Among the PK parameters, K^*trans*^ was found to be the most effective in distinguishing low and high Onco*type*DX risk score ER-positive breast lesions (Fig. 2). While compared to K_*ep*_, K^*trans*^ appeared to have a stronger correlation with the Onco*type*DX risk scores. K_*ep*_ appeared to be more predictive in identifying lesions on DCE-MRI that had a high Onco*type*DX risk score.

### EK Features for Discriminating ER-positive Breast Cancers

The EK features were extracted to quantitatively characterize the contrast enhancement patterns within ROIs in the lesion. Although the uptake rate achieved the best classification performance among all the EK features, these features generally had weaker correlation with the Onco*type*DX risk scores compared to the TK features.

### IK and TK Features for Discriminating ER-positive Breast Cancers

The TK features outperformed the IK features by up to 10% in terms of Az and NPV (Table 1). Further, the TK features had a higher degree of positive correlation with the Onco*type*DX risk categories compared to the IK features which had a lower degree of negative correlation. These trends are consistent with the performance of the classifier (Table 1).

### DHoG and DLBP Features for Discriminating ER-positive Breast Cancers

Both DHoG and DLBP yielded good discrimination for separating lesions corresponding to the low and high Onco*type*DX risk categories, achieving the highest values of Az, PPV, and NPV among all computer extracted MRI features. The DHoG and DLBP features were significantly correlated with the Onco*type*DX risk categories.

**Figure 3:**
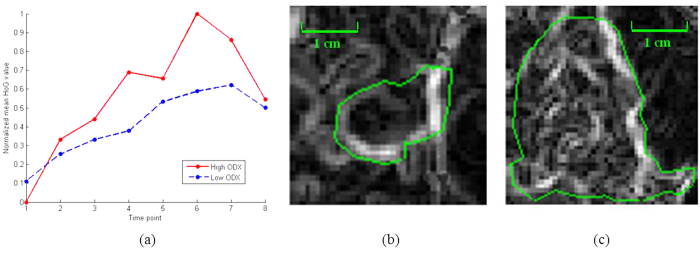
Comparison of contrast enhancement pattern and dynamic histogram of oriented gradient (DHoG) features (4 bins) of estrogen receptor (ER)-positive breast lesions between low Onco*type*DX recurrence score (=15), moderate grade in 49-year-old woman and high Onco*type*DX recurrence score (=40), high grade in 64-year-old woman. (**a**) Normalized mean DHoG values versus time points. (**b**) DHoG feature map of low Onco*type*DX at peak enhancement (7th phase, 1.5T). (**c**) DHoG feature map of high Onco*type*DX at peak enhancement (6th phase, 1.5T). The green contour indicates tumor boundary. Note that the two curves have distinct enhancement patterns. Feature maps associated at peak enhancement reflect great intensity variance between two tumors.

**Figure 4:**
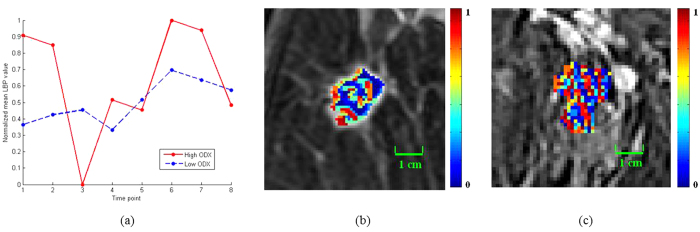
Comparison of contrast enhancement pattern and dynamic local binary pattern (DLBP) features (256 bins) of estrogen receptor (ER)-positive breast lesions between low Onco*type*DX recurrence score (=11), low grade in 53-year-old woman and high Onco*type*DX recurrence score (=41), high grade in 48-year-old woman. (**a**) Normalized mean DLBP values versus time points, and the color-coded DLBP image of (**b**) low Onco*type*DX at peak enhancement (6th phase, 1.5T), and (**c**) high Onco*type*DX at peak enhancement (6th phase, 1.5T). Note that the enhancement patterns vary widely in contrast uptake from time point to time point between two tumors.

**Figure 5:**
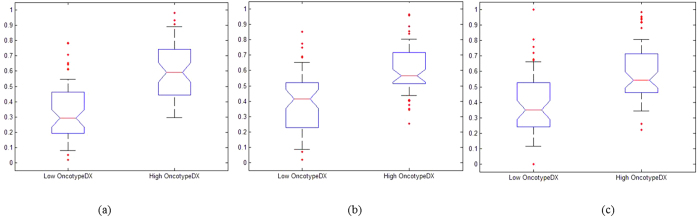
Box-and-whisker plots for mean feature values of three best features corresponding to (**a**) dynamic histogram of oriented gradient (DHoG), (**b**) dynamic local binary pattern (DLBP), and (**c**) pharmacokinetic (PK) feature across all patient studies. The plots suggest that DHoG and DLBP have improved separability between low versus high Onco*type*DX estrogen receptor (ER)-positive breast tumors compared to the PK features.

Figures 3 and 4 illustrate the textural kinetic curves corresponding to contrast uptake and enhancement appearance of the DHoG and DLBP features for representative low and high Onco*type*DX risk breast lesions. The average features values of the top DHoG and DLBP features obtained across all patient studies are shown in Fig. 5(a,b) in comparison with the PK features (Fig. 5(c)).

### Performance of the Feature Combination for Discriminating ER-positive Breast Cancers

The top performing features from each of the 4 classes, including PK (K^*trans*^), TK (Energy, Sobel x-direction gradient), DHoG (4-bin, 6-bin), and DLBP (256-bin), were combined using a linear discriminant classifier. The combined LDA classifier was able to differentiate between low and high Onco*type*DX ER-positive breast cancers with an Az of 0.87 (95% confidence interval: 0.78, 0.96), PPV of 0.76 (95% confidence interval: 0.68, 0.84), NPV of 0.89 (95% confidence interval: 0.83, 0.95), RSD of 5.75, and *err* of 0.147.

### Stability and Predictive Performance

The stability and error of LDA classification were measured by the relative standard deviation and the inverse power law model. It can be observed that DHoG and DLBP outperformed the other feature classes (i.e., shape, PK, EK, TK, IK) and achieved the smallest RSD values and error rates. The IK feature (1^*st*^ fitting coefficient) has the highest RSD values indicating the lowest stability in classification. The shape features produced the largest error rates among all the feature classes.

## Discussion

We presented a computerized image analysis framework for identification of breast MR imaging markers to distinguish between low and high risk ER-positive breast cancers via a correlation of computer extracted DCE-MRI attributes and the Onco*type*DX assay. Although tumor margin, tumor size, rim enhancement on DCE-MRI have been previously correlated with pathological factors and have been reported to be associated with disease outcome^11^,^12^,^13^,^14^,^23^, to the best of our knowledge, this is the first attempt to investigate the association between textural kinetic features on DCE-MRI with Onco*type*DX recurrence scores for ER-positive breast cancers. This is important because the Onco*type*DX is an assay with proven clinical utility that has been shown to be both prognostic and predictive in ER-positive breast cancers^24^. Hence by demonstrating computer extracted imaging features on DCE-MRI can predict the Onco*type*DX risk category of the lesion, we might be able to non-invasively identify which patients would benefit from adjuvant therapy. This could pave the way for non-invasive risk assessment of the lesion even prior to biopsy. Furthermore, this is the first systematic comparison of various kinetic (PK, EK, IK, TK, DHoG and DLBP) and shape features, to discriminate high and low Onco*type*DX categories of ER-positive breast lesions.

There has been recent interest in identifying radiogenomic correlates of breast lesions on MRI. In^16^, Agner *et al.* showed that textural kinetic features extracted from routine clinical DCE-MRI appeared to be associated with the biologic heterogeneity and molecular subtype of breast cancers. Giger *et al.*^25^,^26^ computed enhancement kinetic features, such as time to peak, uptake rate, maximal uptake, from a characteristic time curse curve to distinguish benign and malignant breast masses. Ashraf *et al.*^9^ also utilized curve-based kinetic features to construct breast DCE-MRI phenotypes and showed their correlation with the Onco*type*DX assay.

In this study, we attempted to identify whether there was an association between textural kinetic features extracted from ER-positive breast lesions on 1.5 Tesla DCE-MRI and their corresponding Onco*type*DX risk categories. While Ashraf *et al.*^9^ focused on the association between lesion shape, contrast kinetic features and spatial heterogeneity features and the continuous Onco*type*DX recurrence scores, our approach was focused on evaluating the ability of quantitative image features and spatio-temporal patterns within the lesion to distinguish between the low (<18 risk score) and high (>30 risk score) Onco*type*DX risk categories. Additionally in conjunction with a LDA classifier, the textural kinetic features yielded an Az = 0.84 in distinguishing low and high Onco*type*DX risk category lesions, compared to Ashraf *et al.*^9^ where the Az was 0.77.

Our approach was also different from that of Agner *et al.*^8^ in that we employed two new textural kinetic features, DHoG and DLBP, which unlike EK, IK and TK features, seek to capture contextual textural changes during contrast uptake by considering changes in spatial intensity patterns within divided grid cells in the lesion ROI. Unlike the approach in Agner *et al.*^16^ which attempted to fit a single parametric curve to characterize the temporal changes in lesion texture, the DHoG and DLBP approaches capture the frequency of occurrence of different spatio-temporal textural patterns within the lesion.

A systematic and quantitative analysis of different computer extracted features demonstrated that curve-based kinetic features (i.e., EK, TK, IK) were less discriminating compared to the other three feature classes (i.e., DHoG, DLBP, PK) in distinguishing high and low Onco*type*DX risk score ER-positive cancers. Consistently, the feature combination identified through the feature selection process contained the important features from 4 feature classes (DHoG, DLBP, PK, and TK). While the PK features showed moderate correlation, lesion shape features were even less correlated with the Onco*type*DX risk categories for the lesions evaluated. The DHoG and DLBP appeared to be the most discriminative features in differentiating low and high Onco*type*DX risk score ER-positive breast lesions on DCE-MRI. Figures 3 and 4 which show the normalized mean DHoG and DLBP curves plotted as a function of contrast uptake, appear to illustrate a high degree of heterogeneity in high Onco*type*DX risk score cancers compared to low Onco*type*DX risk score cancers. The corresponding color-coded DHoG and DLBP feature maps at peak enhancement (Figs 3 and 4) also suggest that high Onco*type*DX risk score breast cancers may appear to be more heterogeneous at peak contrast compared to low Onco*type*DX risk score cancers. The Spearmen’s rank correlation test showed that DHoG and DLBP are significantly correlated (DHoG: *ρ* = 0.85, *P* < 0.01; DLBP: *ρ* = 0.83, *P* < 0.01) with the high and low Onco*type*DX risk score categories. These results are consistent with the findings of Ashraf *et al.*^9^, who showed that DCE-MRI based heterogeneity kinetic features were correlated with Onco*type*DX recurrence scores (*ρ* = 0.71, *P* < 0.001). However unlike Ashraf *et al.* where image data from only a single institute was considered, our approach included image data from two different clinical sites.

Our study did have its limitations, and as such, it is important to acknowledge that this is a preliminary study with need for additional independent validation of our initial findings. Additionally, we only included those patients having low (<18) and high (>30) Onco*type*DX recurrence scores and excluded intermediate risk scores (>18 and <30) as the contrast was greatest between these categories and further work is needed to evaluate the intermediate category. Further, the extracted features were computed based on the automated segmentation method due to lack of precise lesion boundary for the data from Site II. Owing to the limited size of the dataset considered in this study, we did not conduct multiple statistical tests of comparisons on the features. We also did not explicitly quantify the inter-observer variability in segmentation of the dominant masses between multiple readers. One problem was the fact that we were identifying imaging markers correlated with a surrogate of outcome (Onco*type*DX) instead of actual outcome itself- unfortunately this information was not available for the patients considered in this study.

### Concluding Remarks

We identified a set of computer extracted image texture features on DCE-MRI that appear to be able to segregate high and low Onco*type*DX risk scores in ER-positive breast cancers. The texture features so identified may allow for non-invasively predicting which ER-positive patients might benefit from adjuvant hormonal and chemotherapy.

## Materials and Methods

This study was approved by the institutional review board and compliant with Health Insurance Portability and Accountability Act. Written informed consent was obtained from all subjects. The experimental protocols were approved by the Case Western Reserve University Faculty of Biomedical Engineering Ethics Committee. The methods were carried out in accordance with the approved guidelines and regulations.

### Patients

**Figure 6:**
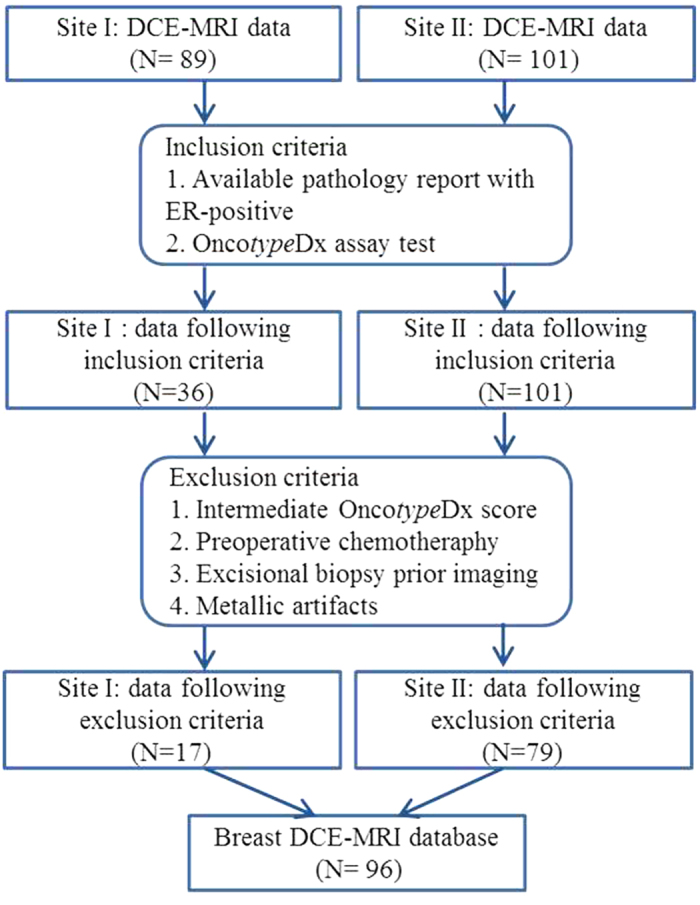
Flowchart of our study population with the patient inclusion and exclusion criteria.

The breast DCE-MRI data were retrospectively collected from two institutions (Site I: Boston Medical Center; Site II: UH MacDonald Women’s Hospital) between 2006 and 2012. All the cases were anonymised. In Site I, women patients who presented with a suspicious breast lesion on screening mammogram, then had diagnostic MRI, were recruited to a large study of MRI in the staging, diagnosis, and screening of breast cancer. In Site II, women whose pathology revealed node-negative, ER-positive invasive breast cancer who participated in a large breast cancer case-control study were utilized for this study^27^. All the DCE-MRI images at 1.5T were obtained within 3–7 days after diagnostic biopsy. The lesion diagnosis for both cohorts was confirmed by ultrasound guided core needle biopsies or MRI guided biopsies, followed by the histopathologic examination of 3–10 specimens obtained by core biopsy sampling. A total of 89 patient studies were collected from the Site I, and only 17 patients with both pathology reports and available low or high Onco*type*DX scores were included in this study. From the Site II, we acquired 101 ER-positive stage I-III female breast cancer patients. Of those, 79 patients had both associated pathology reports and available low and high Onco*type*DX recurrence scores. Patients with intermediate Onco*type*DX recurrence scores (18–30) were deemed to not be informative of cancer risk and hence excluded from the analysis. Three patient studies and one patient study from the Site I had two and three separate lesions respectively. All other patients only had a single lesion. For each patient, the Onco*type*DX test was performed for the dominant mass (index lesion), hence only the index lesion in the case of the patient with multiple masses was considered. The patient selection criteria for our study are summarized in Fig. 6.

### DCE-MRI

Of the 96 patients for whom pathology reports and Onco*type*DX results were available, 17 breast MRIs were acquired from the Site I as multiplanar T1- and T2-weighted images on a 1.5T magnet with an 8-channel breast coil (Achieva; Philips, Best, The Netherlands). The imaging parameters for DCE-MRI were: matrix size, 252 × 286; in-plane resolution, 0.20C0.70 mm per pixel; number of temporal positions: 5–10; echo train length: 50/60; section thickness, 1.5 mmC4 mm; 4.9C7.8/2.3C4.9 [repetition time msec/echo time msec]; flip angle, 10°C30°. The remaining 79 patient studies were acquired from Site II with T1-weighted images using a 1.5T unit (MAGNETOM Avanto; Siemens, Berlin, Germany), and the imaging parameters for DCE-MRI were: matrix size, 230 × 320 C 269 × 384; in-plane resolution, 0.40C0.80 mm per pixel; number of temporal positions: 6C8; echo train length: 50; section thickness, 1.0 mmC2.5 mm; 4.7C8.1/1.5C4.5 [repetition time msec/echo time msec]; flip angle, 10°C25°. Both DCE-MRI data sets were obtained prior to, during, and after administration of 0.1 mmol/kg body weighted of gadolinium-DTPA at a flow of 4cc/second, for a total imaging duration of 5–10 minutes. Each patient study was accompanied by: i) private health information free clinical metadata containing clinical history, age range, and radiology report; ii) pathological reports containing ER-positive scale values denoting low (17–34%), moderate (34–50%), or high (50–100%); and iii) recurrence score denoting lower relapse rate and improved overall survival with adjuvant tamoxifen (<18), or the converse (>30) outcome. Table 2 summarizes the patient characteristics.

### Lesion Segmentation and Feature Extraction

For each patient study, a representative section of the DCE-MRI volume, containing the largest diameter of the dominant mass, was chosen by a radiologist (B.N.B or D.P, both with more than 10 years of experience in the interpretation of breast MRIs) who was blinded to pathologic diagnosis. The lesion boundary was automatically delineated via an automated lesion segmentation method specifically developed and evaluated on breast DCE-MRI^28^. The computer derived features, including shape features, pharmacokinetics (PK), enhancement kinetics (EK), intensity kinetics (IK), TK, DHoG, and DLBP, were calculated based on the pixels enclosed by the delineated regions of interest (ROI) containing breast masses. A flowchart demonstrating the use of computerized features for lesion class discrimination is shown in Supplementary Figure S1 online. Table 3 describes the extracted features. All feature calculations were performed by using software developed in-house and was implemented using the MATLAB^©^ programming platform (version R2013a, MathWorks, Natick, MA).

#### Shape features

Six shape features^8^ were included: (a) area overlap ratio, (b) variance of distance ratio, (c) compactness, (d) smoothness, (e) normalized average radial distance ratio, and (f) standard deviation of normalized distance ratio. These attributes were used to measure the roundness, smoothness, spiculation, and regularity of the lesion margin.

#### Pharmacokinetics

Toft’s PK model^29^,^30^ is most commonly used in DCE-MRI to provide a physiologic interpretation of the breast MRI images via three parameters^31^, i.e., K^*trans*^ (the transfer constant between the plasma and tissue compartments), 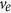 (the extracellular extravascular volume fraction), and K_*ep*_ (the ratio of K^*trans*^/*v*_*e*_). The PK parameters were estimated on the MRI dynamic signal enhancement curves plotted as a function of time after a bolus injection of Gd-DTPA.

#### Enhancement Kinetics

Breast lesion enhancement can be qualitatively characterized by assessing the enhancement curve obtained by plotting the signal intensity values over time after contrast injection. The mean signal intensity at each time point was calculated on the entire lesion ROI. A total of four intensity kinetic features (maximal uptake, time to peak, uptake rate, and washout rate) were computed to measure the amount and rate of contrast uptake^25^,^26^.

#### Intensity Kinetics and Textural Kinetics

A third-order polynomial was fitted to the enhancement curve to characterize its shape via a set of four model coefficients^16^. For each lesion, we computed five types of textural features, including Kirsch, Sobel, Haralick, and first-order textural features. Table 3 summarizes all the textural features considered in this study. The mean textural feature of lesion ROI was plotted as a function of time during the period of contrast administration. These polynomial coefficients represent the corresponding intensity and textural kinetic behavior of the lesion and represent the corresponding IK and TK features.

#### Dynamic Histogram of Oriented Gradient Features

We computed a multi-grid based DHoG at each phase or time point during the DCE-MRI exam. First, a gradient image at each phase was obtained via a gradient filter applied to both horizontal and vertical directions of the ROI containing the lesion. The gradient image was divided into a sequence of increasingly finer spatial grids by repeatedly doubling the number of divisions in each direction. For each grid cell, we calculated the cell histograms by counting the number of occurrences of gradient values in the histogram channels that were evenly distributed from 0 to 360 degrees. An orientation histogram was obtained by aggregating all the cell histograms. The DHoG features for the MRI time series were then obtained by averaging the orientation histograms over the course of different phases. More details on the DHoG features are described in Supplementary-A (online).

#### Dynamic Local Binary Pattern Features

Similar to the computation of the DHoG features, the lesion ROI was divided into multiple grid cells. For each pixel in the cell, we compared the pixel value to that of each of its 8 neighbors. This yielded an 8-digit binary number for the pixel under consideration. A cell histogram based on the binary numbers was then computed and normalized. An average cell histogram was calculated across phases. The DLBP features were then extracted by combining all the average cell histograms via a process of matrix concatenation. More details on the DLBP features can be obtained from Supplementary-A (online).

### Linear Discriminant Analysis based Classification via Cross-validation

To determine computer extracted imaging features on DCE-MRI that best discriminated the low from high Onco*type*DX risk categories, the LDA based classification was performed on the individual feature of each feature class (i.e., shape, PK, EK, IK, TK, DHoG, DLBP) and entire feature set containing all the feature classes (176 features in total). A LDA classifier^34^ was trained using the extracted features to classify images with low or high Onco*type*DX via an iterative 2-fold cross-validation scheme. To reduce overfitting, feature selection was performed on the entire feature set via a sequential floating forward based LDA selection method^35^. Further description regarding the theoretical formulation of feature selection problem and LDA classification can be obtained from Supplementary-B online. The important features were identified during the feature selection process were combined with equal weighting and used in conjunction with the LDA classifier. We assume that the condition probability density function with respect to the low and high Onco*type*DX classes is normally distributed with equal class covariance.

### Analysis

#### Statistical Analysis

The Student *t* test was used to verify that there was no tumor size-related bias or age-related bias between low and high Onco*type*DX risk categories (Table 2). To confirm that our classifiers and features were robust to the choice of MRI scanners and clinical sites, we used a paired *t* test to test the null hypothesis that there were no difference in feature values between data acquired from the two sites. A post-hoc power analysis of the 95% confidence interval was performed. The Spearman’s rank correlation tests measured by correlation coefficient (*ρ*) were performed to determine the relationship between the computer extracted features and the low/high Onco*type*DX risk categories. All analyses were performed by using the IBM SPSS software (version 21.0; IBM, Chicago, IL). A value of *P* < 0.05 was considered to indicate a statistically significant difference.

#### Stability of Classification Performance

In the LDA classification, area under the receiver operating characteristic curve (Az), positive predictive value (PPV), negative predictive value (NPV) were used as performance measures for evaluating the discriminability of each of the individual computer extracted features. In order to assess the stability of LDA classifier, the classification was performed via a 2-fold cross validation strategy. We computed the performance measures 100 times and reported the mean values with 95% confidence interval in the results. We employed a stability measure that Parmar *et al.* used to evaluate the performance of classification methods in their recent radiomic work^34^. The classifier stability was empirically quantified using the relative standard deviation (RSD %), which can be defined as:


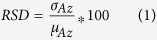


where 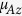 and 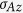 are the mean and standard deviation of the Az values, respectively. The higher RSD values indicate the lower stability in the classification.

#### Stability and Predictive Performance

In order to identify most accurate and highly reliable image features, we used mean values of Az and RSD as feature ranking measures. According to Parmar *et al.*’s selection criterion^32^, the features ranked in the top half of both measures are considered as highly accurate and reliable ones. For each feature class (i.e., shape, PK, EK, IK, TK, DHoG, DLBP), the best identified features have Az greater than the mean Az of all classifiers and RSD less than the mean RSD of all classifiers.

Further, we utilized an inverse power law model of statistical learning to estimate the error rate associated with the classification performance on the currently available data samples. The estimation procedure comprised the following steps: (i) The dataset was divided into a training pool and a testing set via a random sampling; (ii) Ensured that the number of training samples in each set was statistically significant for calculating the power law parameters; (iii) The power law model was applied to describe the relationship between error rate and training set size:


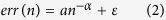


where *err*(*n*) is the error rate for training set size *n*, *a* is the learning rate, *α* is the decay rate, and *ε* is the Bayes error. The model parameters [*a*, *α*, *ε*] can be estimated via a constrained non-linear minimization.

## Additional Information

**How to cite this article**: Wan, T. *et al.* A Radio-genomics Approach for Identifying High Risk Estrogen Receptor-positive Breast Cancers on DCE-MRI: Preliminary Results in Predicting Onco*type*DX Risk Scores. *Sci. Rep.* **6**, 21394; doi: 10.1038/srep21394 (2016).

## Supplementary Material

Supplementary Information

## Figures and Tables

**Table 1:** The best two identified features in each feature class associated with their performance measures in distinguishing low and high risk estrogen receptor (ER)-positive breast cancers.

Feature Class	Feature Name	Az	PPV	NPV	ρ*	*P* value	RSD%	*err*
DHoG	4 bins	0.84 (0.74, 0.94)	0.81 (0.76, 0.86)	0.87 (0.81, 0.93)	0.85 (0.83, 0.87)	0.006	4.76	0.157
	6 bins	0.82 (0.71, 0.93)	0.78 (0.70, 0.86)	0.85 (0.78, 0.92)	0.82 (0.79, 0.85)	0.008	6.09	0.174
DLBP	256 bins	0.80 (0.70, 0.90)	0.74 (0.68, 0.80)	0.85 (0.80, 0.90)	0.83 (0.80, 0.86)	0.008	5.01	0.141
	128 bins	0.79 (0.67, 0.91)	0.74 (0.66, 0.82)	0.83 (0.76, 0.90)	0.83 (0.81, 0.85)	0.013	7.59	0.184
PK	K^*trans*^	0.74 (0.60, 0.88)	0.70 (0.63, 0.77)	0.78 (0.70, 0.86)	0.79 (0.73, 0.85)	0.021	8.11	0.202
	K_*ep*_	0.70 (0.55, 0.85)	0.71 (0.61, 0.81)	0.66 (0.61, 0.71)	0.71 (0.67, 0.75)	0.032	8.57	0.245
EK	Uptake rate	0.72 (0.59, 0.85)	0.63 (0.59, 0.67)	0.74 (0.67, 0.81)	0.65 (0.61, 0.69)	0.064	7.64	0.211
	Time to peak	0.63 (0.52, 0.74)	0.56 (0.51, 0.60)	0.65 (0.57, 0.73)	−0.52 (−0.47, −0.57)	0.212	7.94	0.298
TK	Haralick (Energy)	0.70 (0.57, 0.83)	0.64 (0.59, 0.69)	0.71 (0.63, 0.79)	0.73 (0.70, 0.76)	0.017	7.86	0.257
	Kirsch (Magnitude)	0.68 (0.52, 0.84)	0.60 (0.54, 0.66)	0.72 (0.65, 0.79)	0.72 (0.67, 0.77)	0.052	8.82	0.319
IK	1^*st*^ fitting coefficient	0.64 (0.52, 0.76)	0.60 (0.53, 0.67)	0.64 (0.58, 0.70)	−0.43 (−0.37, −0.49)	0.286	10.16	0.326
	4^*th*^ fitting coefficient	0.63 (0.52, 0.74)	0.58 (0.51, 0.65)	0.64 (0.54, 0.74)	−0.39 (−0.32, −0.46)	0.483	8.73	0.293
Shape	Compactness	0.64 (0.53, 0.75)	0.58 (0.51, 0.65)	0.66 (0.60, 0.72)	−0.57 (−0.52, −0.62)	0.338	7.81	0.334
	Normalized average radial distance ratio	0.60 (0.52, 0.72)	0.53 (0.48, 0.58)	0.67 (0.59, 0.75)	0.53 (0.48, 0.58)	0.502	8.33	0.377

Note. -Numbers in parentheses are 95% confidence intervals.

Az = area under the receiver operating characteristic curve;

PPV = positive predictive value; NPV = negative predictive value;

DHoG = dynamic histogram of oriented gradient; DLBP = dynamic local binary pattern; PK = pharmacokinetics;

EK = enhancement kinetics; TK = textural kinetics; IK = intensity kinetics;

RSD = relative standard deviation; *err* = error rate of classification.

^*^ρ denotes correlation coefficient.

**Table 2:** Characteristics of patients with estrogen receptor (ER)-positive breast cancers.

Parameters	Site I		Site II		*P* value
OncotypeDX recurrence score	Low (<18)	High (>30)	Low (<18)	High (>30)	
No. of Patients (N = 96)	12 (12%)	5 (5%)	43 (45%)	36 (38%)	
Age (y)*	52 (37–68)	47 (36–55)	55 (40–77)	54 (29–70)	0.27
Lesion Size (mm)*	13 (12–30)	21 (7–33)	18 (5–50)	17 (9–40)	0.18
Patient ethnicity					
White	7 (7%)	2 (2%)	36 (38%)	29 (31%)	0.24
African American	3 (3%)	0	7 (7%)	7 (7%)	0.12
Unknown	2 (2%)	3(3%)	0	0	0.08
PR status					
Positive	12 (12%)	3 (3%)	42 (44%)	26 (27%)	0.43
Negative	0	2 (2%)	1 (1%)	10 (11%)	0.21
HER2 status					
Positive	7 (7%)	4 (4%)	18 (19%)	29 (31%)	0.48
Negative	5 (5%)	1 (1%)	25 (26%)	7 (7%)	0.37
Histologic Tumor Grade					
Low	4 (4%)	1 (1%)	10 (11%)	8 (8%)	0.15
Moderate	8 (8%)	2 (2%)	29 (31%)	21 (22%)	0.57
High	0	2 (2%)	4 (4%)	7 (7%)	0.19
Tumor type					
IDC	8 (8%)	3 (3%)	33 (35%)	22 (23%)	0.32
ILC	3 (3%)	0	6 (6%)	11 (12%)	0.16
Mixed	1 (1%)	2 (2%)	4 (4%)	3 (3%)	0.09

Note. -Unless otherwise indicated, data are numbers of patients, with percentages in parentheses.

IDC = invasive ductal carcinoma; ILC = invasive lobular carcinoma.

^*^Data are means, with ranges in parentheses.

**Table 3:** Description of all features used to distinguish low and high risk estrogen receptor (ER)-positive breast cancers.

Feature Class	Lesion Feature	Definition
Shape (k* = 6)	Area overlap ratio	Quantitative measures on lesion shape and lesion margin
	Variance of distance ratio, Compactness, Smoothness	
	Normalized average radial distance ratio	
	Standard deviation of normalized distance ratio	
PK (k = 3)	K^*trans*^	Transfer constant between plasma and tissue compartments
	V_*e*_	The extracellular extravascular volume fraction
	K_*ep*_	The ratio of K^*trans*^/v_*e*_
EK (k = 4)	Maximal uptake, Time to peak	Transfer constant between plasma and tissue compartments
	Uptake rate, Washout rate	
IK (k = 4)	Third polynomial fitting on intensity curve	Intensity kinetic descriptors
TK - first order statistics (k = 48)	Mean, Median	Region intensity statistics derived from lesion area Window size, w 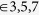
	Range	
	Standard deviation	
TK - Sobel filter (k = 12)	x-direction gradient, y-direction gradient	Edge detectors
	Magnitude of gradient	Window size is 3 × 3
TK - Kirsch filter (k = 36)	Directions: 0, *π*/4, *π*/2, 3*π*/4, *π*, 5*π*/4, 3*π*/2, 7*π*/4	Non-linear edge detector through eight compass directions
	Magnitude of the Kirsch operator	
TK - Haralick (k = 52)	Contrast energy, Contrast inverse moment	Features derived from grey-level co-occurrence matrices
	Contrast average, Contrast variance	
	Contrast entropy	
	Intensity average, intensity variance, intensity entropy	
	Entropy, Energy, Correlation	
	Information Measure 1, Information Measure 2	
DHoG (k = 5)	The number of bins: 2, 4, 6, 8, 10	Histogram based descriptor for gradient orientation on DCE-MRI
DLBP (k = 6)	The number of bins: 8, 16, 32,64, 128, 256	Dynamic local binary pattern features based on texture spectrum

PK = pharmacokinetics; EK = enhancement kinetics; IK = intensity kinetics; TK = textural kinetics;

DHoG = dynamic histogram of oriented gradient; DLBP = dynamic local binary pattern.

^*^k denotes the number of features.
