# Supplementary material for: A Radio-genomics Approach for Identifying High Risk Estrogen Receptor-positive Breast Cancers on DCE-MRI: Preliminary Results in Predicting OncotypeDX Risk Scores
Source: Sci Rep. 2016 Feb 18;6:21394. doi: 10.1038/srep21394 (PMC4757835; doi:10.1038/srep21394)
Supplement: Supplementary Information [file srep21394-s1.pdf]

A Radio-genomics Approach for Identifying High Risk Estrogen Receptor-positive Breast Cancers on DCE-MRI: Preliminary Results in Predicting Onco*type*DX Risk Scores

Tao Wan[1,2], PhD, B. Nicolas Bloch[3], MD, Donna Plecha[4], MD, Cheryl L.Thompson[5], PhD, Hannah Gilmore[6], MD, Carl Jaffe[3], MD, Lyndsay Harris[7], MD, Anant Madabhushi[2], PhD

[1] School of Biological Science and Medical Engineering, Beihang University, Beijing, China

[2] Dept. of Biomedical Engineering, Case Western Reserve University, OH, USA

[3] Dept. of Radiology, Boston University School of Medicine, MA, USA

[4] Division of Breast Imaging, UH MacDonald Women's Hospital Breast Centers, OH, USA

[5] Dept. of Epidemiology and Biostatistics, Case Western Reserve University, OH, USA

[6] Division of Anatomic Pathology, Case Western Reserve University, OH, USA

[7] Division of Hematology and Oncology, Seidman Cancer Center, OH, USA

Corresponding author:
Tao Wan, PhD
School of Biological Science and Medical Engineering, Beihang University
37 XueYuan Road
Beijing, China, 100191
Phone:+86(01)82316960
Fax:+86(01)82316960
Email: tao.wan.wan@gmail.com

Supplementary A: Feature Descriptions (DHoG and DLBP)

The histogram of oriented gradients (HoG)[1] and local binary patterns (LBP)[2] descriptors are two popular features that are extensively used in object detection and pattern recognition. Previous studies showed that the LBP and HoG features yielded high accuracy in detecting breast masses on mammographic images[3,4]. Although the LBP and HoG showed promise in various applications in computer vision and image processing, they have not been fully explored in the medical imaging filed. Both features are powerful descriptors to characterize local attributes of images, thus making them as favorable imaging attributes to precisely describe subtle changes inside breast lesions in dynamic contrast enhanced (DCE)-MRI as well as small differences between estrogen receptor (ER)-positive breast cancers. In this study, two new features, i.e., the dynamic local binary patterns (DLBP), and dynamic histogram of oriented gradients (DHoG), based on the popular LBP and HoG features, were computed to characterize the imaging changes inside breast lesions over the course of contrast administration.

We define 2D sections of 3D MRI volumes as $\mathrm{C} = (\mathrm{C}, f^t)$, where C is a 2D set of pixels $\mathrm{c} \in \mathrm{C}$, and f^t is the associated intensity function at every pixel c at each time point t $\in \{0,1, \dots, \mathrm{T}-1\}$ in the DCE-MRI time series. $\mathrm{C} = (\mathrm{C}, f^0)$ refers to the pre-contrast image.

DHoG Features

Since the HoG descriptor divides the image into blocks and these blocks are overlaid, we computed a multi-scale based HoG features[5] on each time point MRI. First, a gradient image $\mathrm{G}(f^t)$ was obtained via a gradient filter $[-1,\ 0,\ 1]$ applied on both horizontal and vertical directions of $\mathrm{G}(f^t)$. The gradient image $\mathrm{G}(f^t)$ was divided into small cells at resolution scale s, $\mathrm{s} \in \{1, \dots, \mathrm{S}\}$. Each pixel c in examined cell r_j^s, $\mathrm{j} \in \{1, \dots, \mathrm{V}\}$, calculated a weighted vote w_c based on the orientation of the gradient element centered on it. An orientation histogram $h^s\big(G(f^t)\big) = [h(r_1^s), \dots, h(r_V^s)]$ was obtained by accumulating each w_c for $\mathrm{c} \in r_j^s$, $\mathrm{j} \in \{1, \dots, \mathrm{V}\}$, $\mathrm{s} \in \{1, \dots, \mathrm{S}\}$. The DHoG feature for DCE-MRI time series can be computed as:

$$\mathrm{F}_{DHoG}^{b} = [\bar{h}^1, \dots, \bar{h}^S]$$

where $\bar{h}^s = \frac{1}{T}\sum_{t=0}^{T-1} h^s(G(f^t))$, and *b* is the number of orientation bins.

DLBP Features

For $\mathrm{C}(f^t)$ at time point t, breast lesion L was divided into multiple cells g_i, $\mathrm{i} \in \{1, \dots, \mathrm{U}\}$. For each pixel $\mathrm{c} \in g_i$, compared the pixel to each of its 8 neighbors, which gave an 8-digit binary number for this pixel. A histogram $h_{g_i}^t$ over the cell was then computed and normalized. A mean histogram $\bar{h}_{g_i} = \frac{1}{T}\sum_{t-0}^{T-1} h_{g_i}^t$ was calculated across time points. The DLBP can be formed by:

$$\mathrm{F}_{DLBP}^{b} = \left[\bar{h}_{g_i}, \dots, \bar{h}_{gU}\right]$$

where $[\cdot]$ is a matrix concatenation, and *b* is the number of bins used in the histogram.

Supplementary B: Feature Selection and Classification

Linear Discriminant Analysis (LDA) based Feature Selection

We used a sequential floating forward based LDA feature selection method[6]. In this algorithm, addition and removal of features is repeated alternately in a stepwise manner. In general, after addition of each feature, there is a back-track loop in which features are removed from the set. The feature to be added or removed is always the feature that yields a better performance than all subsets obtained when one of the other features was added or removed instead.

Linear Discriminant Analysis Classification

The linear discriminant analysis classifier[7,8] was trained using the computer extracted features to classify breast tumors with low or high Onco*type*DX recurrence scores via a *2*-fold cross-validation scheme. In the LDA classification, we assumed the condition probability density function $\mathrm{p}(\mathrm{F}(\mathrm{C})|\omega_i)$, $\mathrm{i} \in \{1{,}2\}$, where ω_1 and ω_2 represented the low and high Onco*type*Dx categories, respectively, was normally distributed with equal class covariances. The LDA classifier can be defined as:

$$\tilde{l} = \underset{l=\omega_1,\omega_2}{\arg\min} \sum_{i=1,2} p\big(\omega_i \big| F(C)\big)\psi(l|\omega_i)$$

where $\tilde{l}$ is the predicted label, $p(\omega_i|s)$ is the posterior probability of class ω_i for the feature set, and $\psi(l|\omega_i)$ defines the cost of classifying an observation as l when its true class is ω_i. The classifier was implemented in using the statistics and machine learning toolbox of MATLAB© programming platform.

Supplementary Figures

Figure S1. The flowchart shows workflow of lesion classification in distinguishing low and high Onco*type*DX risk estrogen receptor (ER)-positive breast cancers addressed in this study. Following the automated lesion segmentation, 7 different feature classes (shape, enhancement kinetics, intensity kinetics, pharmacokinetic, textural kinetics, DLBP, DHoG) were computed. Linear discriminant analysis (LDA) was used to identify the feature set that enabled the most discrimination between the low and high Onco*type*DX risk categories.

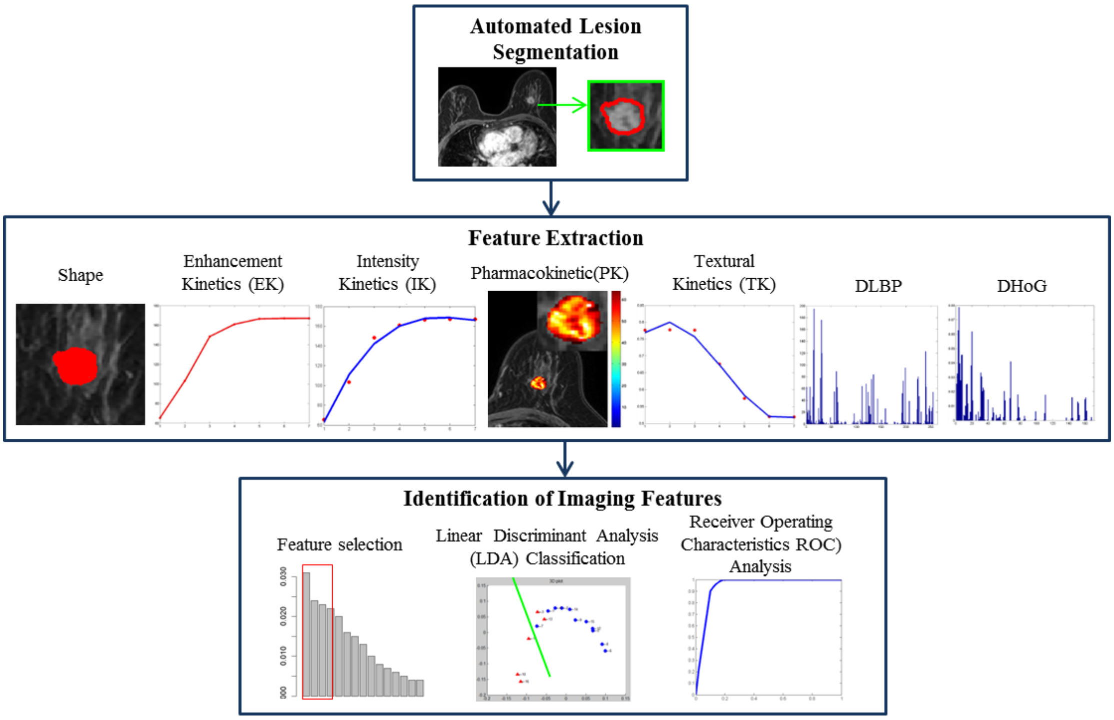

Supplementary Tables

Table S1. The values of best two identified features in each feature class and the top performing 6 features (K^{trans}, Energy, Sobel x-direction gradient, DHoG 4-bin, DHoG 6-bin, DLBP 256-bin shown in bold) were combined using a linear discriminant classifier.

Feature Class	Feature Name	Low Onco*type*DX (<18, N=55)	High Onco*type*DX (>30, N=41)
DHoG	**4 bins**	**43.71±92.08**	**96.40±53.31**
	**6 bins**	**71.88±69.14**	**117.99±43.22**
DLBP	**256 bins**	**80.90±50.38**	**124.14±36.60**
	128 bins	61.08±73.03	99.65±58.94
PK	$\boldsymbol{K^{trans}}$	**0.33±0.41**	**0.58±0.53**
	K_{ep}	0.17±0.55	0.29±0.49
EK	Uptake rate	6.76±8.77	12.42±5.21
	Time to peak	5.87±2.16	3.84±3.92
TK	**Haralick (Energy)**	**0.10±0.54**	**0.17±0.46**
	Kirsch (Magnitude)	0.74±3.96	0.80±3.87
IK	1st Fitting coefficient	10.85±33.63	6.01±51.45
	4th Fitting coefficient	108.54±70.14	88.24±101.30
Shape	Compactness	-19.05±9.14	-20.76±7.32
	Normalized average radial distance ratio	0.76±0.23	0.80±0.15
Feature combination	**Sobel x-direction gradient**	**0.32±3.38**	**0.56±5.46**

Note.—Data are means ± standard deviations

References

1. Dalal N. & Triggs B. Histograms of oriented gradients for human detection. *CVPR* 886–893 (2005).
2. Ojala T., Pietikainen M. & Harwood D. Performance evaluation of texture measures with classification based on kullback discrimination of distributions. *ICPR* 582–585 (1994).
3. Berbar M.A., Reyad Y.A. & Hussain M. Breast mass classification using statistical and local binary pattern features. *ICIV* 486–490 (2012).
4. Kage A., Elter M. & Wittenberg T. An evaluation and comparison of the performance of state of the art approaches for the detection of spiculated masses in mammograms. *EMBS* 3773–76 (2007).
5. Bosch A., Zisserman A. & Munoz X. Representing shape with a spatial pyramid kernel. *CIVR* 401–08 (2007).
6. Hupse R. & Karssemeijer N. The effect of feature selection methods on computer-aided detection of masses in mammograms. *Phys. Med. Biol.* **55**, 2893-2904 (2010).
7. Martinez A. M. & Kak A. C. PCA versus LDA. *IEEE Trans. Pattern Anal. Mach. Intell.* **23**, 228-233 (2001).
8. Duda R. O., Hart P. E. & Stork D. G. *Pattern Classification 2nd edn*, Ch. 5, 215-281 (John Wiley & Sons, New York, USA, 2001).
